# A century of winter wheat breeding decreases root hydraulic traits

**DOI:** 10.1093/plphys/kiaf235

**Published:** 2025-06-06

**Authors:** Yunji Huang, Ning Zhang

**Affiliations:** State Key Laboratory of Wheat Improvement, Shandong Agricultural University, Tai'an, Shandong 271018, China; State Key Laboratory of Wheat Improvement, Shandong Agricultural University, Tai'an, Shandong 271018, China

As 1 of the 3 main grain crops worldwide, wheat supplies approximately 20% of both the daily caloric requirements and protein intake for the global human population (Tadesse et al. 2019). Therefore, breeding high-yielding wheat cultivars is essential to meet the growing demand. Historically, breeders usually selected cultivars based on aboveground traits and yield, while root traits are overlooked, partly due to the technical challenges in root phenotyping (Waines and Ehdaie 2007). The root system plays a pivotal role in plant growth and productivity through its water and nutrient absorption, transport, and environmental adaptation. Importantly, these functions are determined by the root hydraulic architecture, a system that integrates root architecture and hydraulics (Maurel and Nacry 2020). Among root traits, root system conductance (*K*_rs_), a key component of root hydraulics, governs the capacity of water uptake by roots (Baca Cabrera et al. 2024). Although several studies have demonstrated that root architecture has been altered by modern breeding programs (McGrail and McNear 2021), there remains limited understanding of how wheat root traits have co-evolved with aboveground traits targeted by breeders, particularly regarding modifications in *K*_rs_.

Recently in *Plant Physiology*, Baca Cabrera et al. (2025) shed light on the unintentional selection pressures exerted by breeders on wheat root morphology and hydraulic traits over the past century, within the context of evolving climate conditions and agricultural cultivation practices. The researchers conducted field measurements of root architectural traits, including seminal (embryonic primary root), crown (post-embryonic nodal roots), and lateral roots (branching secondary roots), in 6 German winter wheat cultivars released at various times over the past century: (1) S. Dickkopf—1895, (2) SG v. Stocken—1920, (3) Heines II—1940, (4) Jubilar—1961, (5) Okapi—1978, and (6) Tommi—2002. Their analysis revealed a statistically significant century-long decrease in root axis numbers across all root types during modern breeding history. Strikingly, root diameters exhibited no comparable declining trend, with the exception of a marginal decrease observed specifically in lateral roots. These findings are corroborated by similar observations in wheat cultivars from China (Zhu et al. 2019), the UK and Northern Europe (Fradgley et al. 2020), and the US (McGrail and McNear 2021), demonstrating that modern breeding programs across distinct agroecological zones may have driven convergent modifications in root axis numbers without affecting diameters. Surprisingly, the authors found that crown root number strongly positive correlates with tiller (shoot) number, indicating that crown root number is linked to plant size. They also observed that modern cultivars tend to have both fewer crown roots and fewer tillers compared to older cultivars. This reduction in crown roots and tillers may help decrease the competition between plants, potentially making modern cultivars better suited for high-density planting (Fang et al. 2011).

Moreover, laboratory measurements of *K*_rs_ in young seedlings cultivated under hydroponic conditions revealed a significant decrease in the most modern cultivar compared to the oldest cultivar, with values declining from 1.3 × 10^−10^ to 0.7 × 10^−10^ m^3^ MPa^−1^ s^−1^. A comparable yet attenuated trend emerged when normalizing *K*_rs_ by root system surface area (*K*_rs_area_) or total length (*K*_rs_length_). To extrapolate *K*_rs_ dynamics to later developmental phases, the authors applied the whole-plant architectural model CPlantBox to 2 historically contrasting cultivars (oldest vs newest), simulating the coordinated progression of *K*_rs_ with plant growth. The simulation curves of both cultivars exhibited that *K*_rs_ increased dramatically during the initial 20- to 30-day developmental phase as a consequence of root growth, followed by a gradual decline in its elevation rate until reaching relative stability at the simulation endpoint. Notably, simulated *K*_rs_ remains persistently higher in the oldest cultivar compared to the newest cultivar, which was consistent with the measured *K*_rs_ values. Combined with the aforementioned finding that modern cultivars generally exhibit smaller root system dimensions, these results suggest that contemporary breeding strategies may favor genotypes with diminished root water uptake capacity. In rainfed agroecosystems—such as those common in Germany, where the study was conducted—cultivars with decreased *K*rs may adopt a more water-conservative strategy. This diminished root water uptake capacity can help conserve soil moisture during early growth stages, potentially allowing for more efficient water use during critical periods such as reproduction or drought stress later in the season (Passioura 1972; Richards and Passioura 1989).

The study by Baca Cabrera et al. (2025) provides novel insight into the long-term changes in *K*_rs_ associated with wheat breeding. The authors found a decrease in both root axis number and *K*_rs_area_ or *K*_rs_length_ over a century of breeding, leading to a significant decline in *K*_rs_ of wheat (Figure). However, the question remains unsolved whether modern breeding programs across distinct agroecological zones have exerted the convergent selection on *K*_rs_, as the authors conducted *K*_rs_ measurements only on wheat cultivars from Germany. Nonetheless, the findings by Baca Cabrera et al. (2025) provide valuable phenotype information for the selection of investigation materials and for the assistance of wheat breeding. The authors emphasize that the root traits co-selected with aboveground traits encompass not only the decline in root axes number but also the critical *K*_rs_, which may optimize the root architecture, water absorption, and transport at the population level under high-density planting, thereby enhancing resource allocation to grains for improved yield. Although the quantitative trait loci (QTL) associated with root axes number (Atkinson et al. 2015) and a few genes controlling root angels (Fusi et al. 2022) have been identified in wheat, no genetic factors associated with *K*_rs_ have been investigated so far. Future work could identify QTL associated with root hydraulic traits by employing very old versus modern cultivars to construct mapping populations. Also, future research could elucidate the domestication process of root architecture and *K*_rs_ from the insights of root anatomy and genetics while concurrently evaluating the association between *K*_rs_, water use efficiency, and yield during this process.

**Figure.**
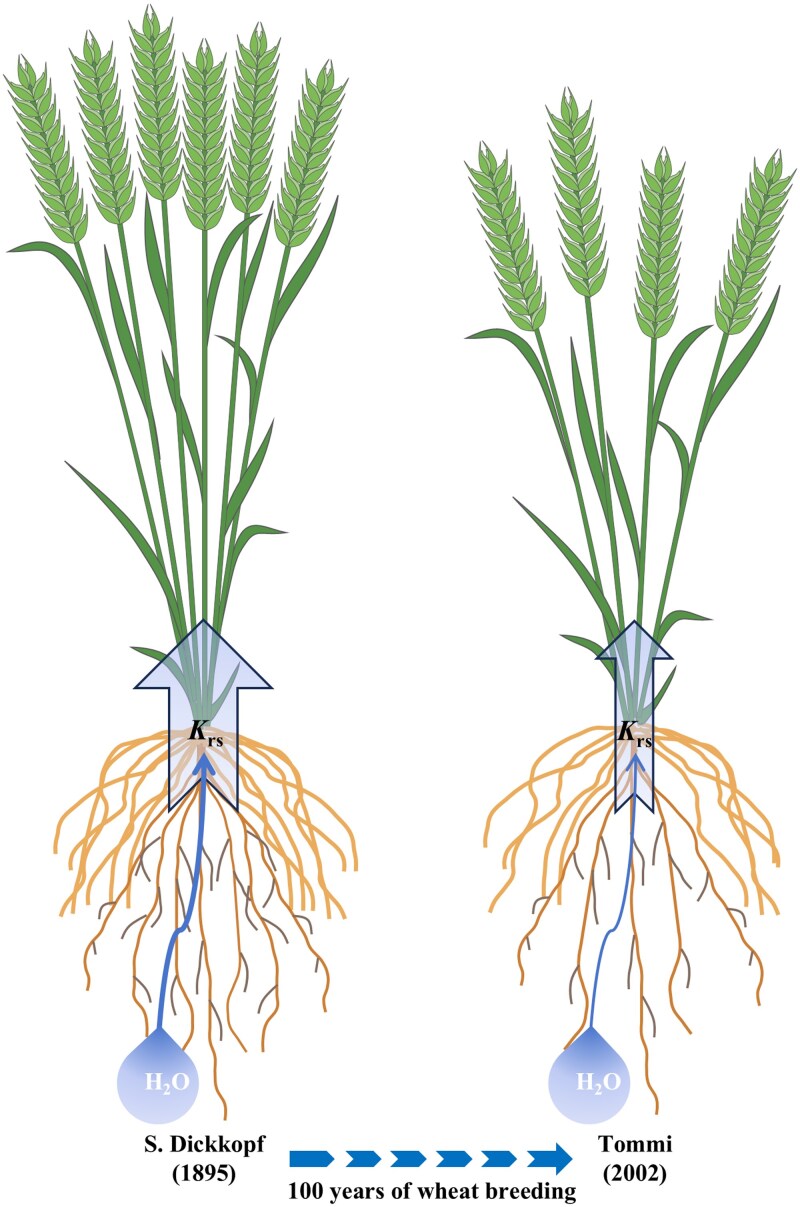
Model depicting the selection exerted by breeders over a century of wheat breeding (summarized from Baca Cabrera et al. 2025). On the left side of the model is the oldest cultivar, which has a larger tiller number and root axis number, including crown roots and seminal roots but excluding lateral roots, and a higher *K*_rs_ compared to the newest cultivar on the right side. Such domestication has endowed modern cultivars with a more conservative root water uptake capability while adapting them to modern high-density planting practices, thereby improving overall yield per unit area.

## Data Availability

No data were generated in this study.
